# Annualized bleeding rate in hemophilia A patients in Brazil: a systematic review

**DOI:** 10.1016/j.htct.2025.103736

**Published:** 2025-03-28

**Authors:** Alessandra NL Prezotti, Débora MC Rocha, Endi L. Galvão, Thaís Gimenez, Leo Sekine, Rodrigo A. Ribeiro, Elisa Sobreira

**Affiliations:** a Centro de Hematologia e Hemoterapia do Espírito Santo, Vitória, ES, Brazil; b Departamento de Fisioterapia, Universidade Federal dos Vales do Jequitinhonha e Mucuri, MG, Brazil; c Departamento de Ortodontia e Odontopediatria da Faculdade de Odontologia da Universidade de São Paulo, SP, Brazil; d Serviço de Hemoterapia do Hospital de Clínicas de Porto Alegre/Faculdade de Medicina da Universidade Federal do Rio Grande do Sul, Porto Alegre, RS, Brazil; e HEMAP Consulting, Porto Alegre, RS, Brazil; f BioMarin Farmacêutica do Brasil, São Paulo, Brazil

**Keywords:** Bleeding disorders, FVIII, Latin America, Prophylaxis

## Abstract

**Background:**

Hemophilia A is an X-linked chronic bleeding disorder due to deficiency of the coagulation factor VIII. According to the residual level of FVIII activity, patients can present with severe (FVIII levels <1 %), moderate (1–5 %) or mild (6–40 %) phenotypes. While long-term prophylaxis is the current standard of care and has been shown to be effective in minimizing bleeding episodes, episodes of hemarthrosis, that could lead to arthropathy and disability, are still reported. This systematic review aimed to evaluate available data concerning current treatment outcomes in severe hemophilia A patients without inhibitors in Brazil, focusing on the frequency of bleeding episodes and adherence to therapy of patients under prophylactic treatment.

**Method:**

A literature search strategy was used in the MEDLINE (via PubMed), Embase, LILACS and SciElo databases from 2014 onwards, since it was the moment that prophylaxis effectively became available in the Brazilian National Health Service, even though prophylactic treatment had been officially incorporated in 2011 focused on concerning bleeding episodes and adherence rate of this population.

**Results:**

Searches yielded 536 articles. After removal of duplicates, 417 articles were screened for eligibility. Eventually, 104 articles were selected for full-text assessment. Finally, only five publications met eligibility criteria and were selected for the descriptive review.

**Conclusion:**

Available information on efficacy of severe hemophilia A management in Brazil currently relies on scarce and possibly biased information. It should be strongly emphasized that Brazil is in great need of a structured and coordinated effort to improve collection, analysis, and reporting of data on hemophilia A patients.

## Introduction

Hemophilia A is an X-linked chronic bleeding disorder due to deficiency of the coagulation factor VIII (FVIII).^1^ Although considered a rare disease, it is possible that numbers have been grossly underestimated,^2^ with previously reported hemophilia A incidence rates at 1 case in 5000 male births,^3^ and an observed prevalence rate of 10.5 patients per 100,000 males.^4^ The estimated worldwide prevalence of patients with hemophilia (both hemophilia A and B) reaches a total of 1,125,000 individuals, while an estimated 418,000 individuals will present severe manifestations of the disease.^5^

Small amounts of residual FVIII activity exert a large clinical impact in hemostasis. Patients with severe deficiency (FVIII levels <1 %) usually fare worse than moderately (1–5 %) or mildly (6–40 %) affected patients.^1^ Indeed, the cornerstone of treatment is replacement therapy, increasing FVIII levels with intravenous injections, either episodically to treat acute bleeding or prophylactically to prevent them.^5^ Long-term prophylaxis is currently standard of care and has been shown to be very effective in minimizing bleeding episodes, especially hemarthrosis, that could lead to arthropathy and disability.^2^ However, due to terminal half-life of traditional FVIII replacement, frequent injections are needed, making it rather burdensome and expensive for patients and the healthcare system, while also compromising treatment access and adherence.^5^

While much effort has been made during the last few years aiming at developing new alternatives for hemophilia A patients such as extended half-life clotting factor concentrates, bispecific monoclonal antibodies (e.g. emicizumab) and gene therapy, patients in Latin America still seem to struggle to attain adequate access to comprehensive multidisciplinary treatment. In Brazil, patients with hemophilia, and several other types of coagulopathies, are managed at blood centers, governmental dedicated healthcare facilities that hold and distribute all clotting factor concentrates. Despite this centralized care, access to contemporary therapeutic options and pipeline drugs and therapies is limited due to cost-effectiveness concerns. Furthermore, clinical data on severe hemophilia A patients have not been adequately summarized, especially after implementation of the 2014 national policy for primary prophylaxis.

## Objective

The present systematic review aimed to evaluate available data concerning current severe hemophilia A treatment outcomes in Brazil, focusing on the frequency of bleeding episodes and adherence to therapy of patients under conventional treatment.

## Methods

The main objective of the present study was to systematically review relevant data on severe hemophilia A management outcomes in Brazil, especially concerning bleeding episodes (annualized bleeding rate [ABR]) and adherence rate of this population.

### Information sources and search strategy

A literature search strategy was performed in the MEDLINE (via PubMed), Embase, LILACS and SciElo databases. No language restrictions were used but the time of publication was restricted to 2014 onwards, since it was the time that prophylaxis effectively became available in the Brazilian National Health Service, even though prophylactic treatment had been officially incorporated in 2011.

The search strategy for each database is shown in Table 1. All searches were restricted to between 2014 and 2022. Overall, the search terms were as follows: population was defined as Brazilian hemophilia A patients; intervention included any type of prophylaxis (whether primary, secondary, or tertiary); the outcomes were ABR and adherence to treatment; and type of study comprised both observational studies and clinical trials.

**Table 1:** Search strategy employed for each database.

Database	Search strategy
PubMed/MEDLINE	((((((((((("Factor VIII deficiencies") OR ("Factor VIII deficiency")) OR ("FVIII deficiencies")) OR ("FVIII deficiency")) OR ("Hemophilia A")) OR ("Haemophilia A")) OR (a, hemophilia[MeSH Terms])) OR (hemophilia)) OR (hemophilia[Title/Abstract])) OR (haemophilia[Title/Abstract])) AND ("bleedings"[All Fields] OR "hemorrhage"[MeSH Terms] OR "hemorrhage"[All Fields] OR "bleed"[All Fields] OR "bleeding"[All Fields] OR "bleeds"[All Fields] OR "prophylaxis"[All Fields] OR "prophylaxes"[All Fields] OR "prophylaxis"[All Fields])) AND ((brasil* or Brazil* or Brazil[ad]))
EMBASE	('bleedings' OR 'hemorrhage'/exp OR 'hemorrhage' OR 'bleed' OR 'bleeding'/exp OR 'bleeding' OR 'bleeds' OR 'prophylaxis'/exp OR 'prophylaxis' OR 'prophylaxes' OR 'prophylaxis') AND ('brasil' OR 'brasileiro' OR 'Brazil'/exp OR 'Brazil' OR 'Brazilian'/exp OR 'Brazilian') AND ('factor viii deficiencies' OR 'factor viii deficiency'/exp OR 'factor viii deficiency' OR 'FVIII deficiencies' OR 'FVIII deficiency' OR 'hemophilia a'/exp OR 'hemophilia a' OR 'haemophilia a'/exp OR 'haemophilia a' OR 'a, hemophilia' OR 'hemophilia'/exp OR hemophilia OR 'haemophilia'/exp OR haemophilia)
Lilacs	'factor viii deficiencies' OR 'factor viii deficiency' OR 'FVIII deficiencies' OR 'FVIII deficiency' OR 'hemophilia a' OR 'hemophilia a' OR 'haemophilia a' OR 'haemophilia a' OR 'a, hemophilia' OR 'hemophilia'/exp OR hemophilia OR 'haemophilia' OR haemophilia [words] and Brazil OR Brazil [words]
Scielo	factor viii deficiencies OR factor viii deficiency OR FVIII deficiencies OR FVIII deficiency OR hemophilia a OR hemophilia a OR haemophilia a OR haemophilia a OR a, hemophilia OR hemophilia/exp OR hemophilia OR haemophilia OR haemophilia

Duplicates were excluded before proceeding to study selection. All titles and abstracts retrieved were screened independently by two researchers. Full-text articles also had their eligibility evaluated by two independent researchers. The last date of the search was May 18th, 2022. The review protocol was registered in the OSF registries database (https://osf.io/am4pg). This study followed the Preferred Reporting Items for Systematic Reviews and Meta-Analyses (PRISMA) statement for conducting studies and reporting results.

### Eligibility criteria

Observational studies and clinical trials that fulfilled the following criteria were selected: 1) they were concerned with hemophilia A patients with a congenital bleeding disorder resulting from FVIII deficiency; 2) Brazilian patients with severe hemophilia A without inhibitors, receiving some type of prophylactic FVIII; and 3) Prophylaxis could be conceptually primary, secondary, or tertiary. No comparators were required and the main outcome to be evaluated was the reported ABR. Proceedings from major international meetings in the field and letters to the editor were also included. In vitro or animal model studies, review articles, guidelines, qualitative studies, expert opinion articles and case reports were excluded.

### Study selection and data extraction

Two reviewers independently participated in the screening and full-text evaluations. A third reviewer participated in the case of any discordance.

Data were tabulated in Excel spreadsheets (Microsoft Corp, Washington, USA) by the two independent reviewers. A data extraction form included the following information:

Study characteristics: author and year of publication, country, and follow-up period;
Sample characteristics: n, mean age, gender, and treatment status (Y/N); outcomes evaluated;

Main findings: descriptive and quantitative results, effect size, and *p*-value whenever available.

### Quality assessment and risk of bias

The risk of bias was assessed using the Risk of Bias in Non-randomized Studies of interventions (ROBINS-I)^6^. The authors answered signaling questions for each domain (confounding, selection, classification of interventions, deviation from intended interventions, missing data, measurement of outcome, and selection of the reported results). They then estimated the overall risk of the bias according to the results for each domain as low, moderate, serious, or critical. The risk of bias analysis considered studies with a before-after design, without a comparative group.

### Strategy for data synthesis

Descriptive synthesis, and when considered feasible, a meta-analysis with the ABR and adherence rate values were planned.

## Results

**Figure 1:**
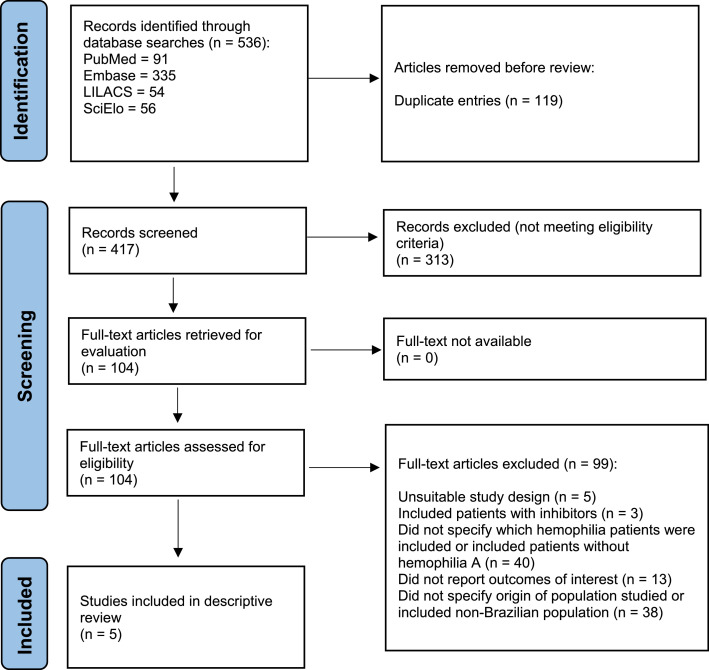
Included studies - flow diagram.

The PRISMA flowchart illustrating the study selection process is shown in Figure 1. The searches yielded 536 records (including duplicate entries). After removal of duplicates, 417 references were screened for eligibility. Eventually, 104 records were selected for full-text assessment. Only five publications^4^^,^^7^, ^8^, ^9^, ^10^ met eligibility criteria and were selected for descriptive review. Meta-analysis of data retrieved could not be performed due to the heterogeneity of the studies.

Data pertaining adherence to prophylactic treatment could not be retrieved according to established selection criteria.

### *Study by* Kenet et al.*^4^*

This was a multinational, prospective, non-interventional study that aimed at collecting standardized real-world data on bleeding episodes, hemophilia medication use, and health-related quality of life (QoL) from a global, heterogeneous population of participants with severe hemophilia A on currently available FVIII prophylaxis. Participating sites were located in Australia, Belgium, Brazil, France, Germany, Israel, Italy, South Africa, South Korea, Spain, Taiwan, the UK, and the US. This study was also a run-in for the sponsor's Phase 3 gene therapy studies (Clinicaltrials.gov NCT03370913/EudraCT 2017–003215–19, NCT03392974/EudraCT 2017–003573–34).

Enrolled patients were males, 18 years of age or older, with severe hemophilia A (FVIII activity ≤1 IU/dL), continuously treated with prophylactic exogenous FVIII for six months or more and no history of detectable FVIII inhibitors. Patients were excluded if they were HIV-positive, had significant liver dysfunction, chronic or active hepatitis B, or active hepatitis C. High-quality historical documentation concerning bleeding and exogenous FVIII usage over the previous six months was required.

Study procedures included a review of bleeding episodes (including start date/time, type [e.g., joint or muscle], location, and whether there was preceding trauma or ensuing treatment), FVIII replacement (start date/time, product name, dose, indication [e.g. usual prophylaxis, one-time prophylaxis, or treatment for bleeding]) at least at a monthly basis (weekly evaluations were recommended whenever possible), as well as the monitoring of concomitant medications, adverse events (AEs), serious AEs (SAEs), and interim medical history at each visit or with telephone calls on at least a monthly basis. Except for screening/baseline and end-of-study visits, all other study visits occurred according to participants’ local standard of care. No clinical intervention or study drug was provided.

The primary clinical endpoint was ABR requiring exogenous FVIII replacement treatment. Secondary endpoints included annualized utilization (IU/kg/year) and infusion rate (count/year) of exogenous FVIII replacement therapy. Also, patient-reported outcomes such as the hemophilia-specific health related quality of life questionnaire for adults (Hemo-QoL-A), EQ-5D-5 L, Hemophilia Activities List (HAL), and Work Productivity and Activity Impairment plus Classroom Impairment Questions: Hemophilia Specific (WPAI+CIQ:HS) were evaluated. Safety assessments consisted of monitoring AEs (coded using the Medical Dictionary for Regulatory Activities v20.1) and measuring vital signs and hematology, clinical chemistry, and urinalysis variables.

A total of 370 patients were screened for eligibility and eventually 294 patients were enrolled. From those enrolled, 225 (76.5 %) completed at least six months of follow up and were included in the six-month analysis population. Results are presented by region, and as the only study site from South America was Brazil, whole data originated from the Hemocentro, a reference tertiary healthcare provider established in the city of Campinas and coordinated by the State University of Campinas. Patient demographics and baseline characteristics for the Brazilian subgroup are found in Table 2. The Brazilian patients had the lowest median age at enrolment (27 years old) while East Asia participants had the highest median age (40 years old). Also, lowest rates of problem joints (defined as joint with chronic pain, chronic synovitis, hemophilic arthropathy, limited motion or recurrent bleeding) were found in Brazilians (9.3 %) while East Asia had the highest rates (56.3 %).

**Table 2:** Patient demographics and baseline characteristics of the Brazilian hemophilia patients.^4^

Parameter	*n* = 54
Age at enrolment (years) - median (min-max)	27.0 (18.0–47.0)
Male sex - n (%)	54 (100.0)
Race - n (%) Black or Afro-American White	10 (18.5)44 (81.5)
Weight (kg) - mean (SD)	78.9 (20.4)
History of hepatitis B^a^ - n (%)	1 (1.9)
History of hepatitis C^a^ - n (%)	12 (22.2)
History of HIV - n (%)	0
Participants with problem joints^b^ - n (%)	5 (9.3)
Number of problem joints^b^ - n (%) 0 1 2 3 >3	49 (90.7)5 (9.3)000

a Includes cleared or cured infections.

b Problem joints were identified by investigators at baseline and were defined as joints with any of the following symptoms: chronic joint pain, chronic synovitis, hemophilic arthropathy, limited motion, or recurrent bleeding HIV: human immunodeficiency virus; SD: standard deviation.

For the six-month analysis, the median follow-up time was 225.0 days (range: 169–469 days). Follow-up time specifically for Brazilian population was not reported. The ABR concerning treated bleeds, for Brazilian patients (*n* = 41) was reported for pre-baseline (mean: 2.44; standard deviation [SD]: 3.83; median: 0.00; range: 0.0–14.0), on-study (mean: 2.41; SD: 4.61; median: 0.00; range: 0.0–23.8), and total study duration (mean: 2.42; SD: 4.05; median: 0.80; range: 0.0–19.3) intervals. As shown, pre-baseline rate was consistent with on-study ABR.

Although no formal comparison was performed by the authors (it is mentioned that the study was underpowered to assess differences between the variables collected), mean and median treated ABR values reported for Brazilian patients seemed lower than the whole population (pre-baseline: mean: 5.03; SD: 9.35; median: 2.00; range: 0.0–86.0]; on-study: mean: 4.33; SD: 6.39; median: 1.85; range: 0.0–37.8; total study duration: mean: 4.64; SD: 7.00; median: 2.27; range: 0.0–57.8). Data for all bleeding events and stratified by treated bleed categories (whether spontaneous, traumatic, joint bleeds and problem joint bleeds) was not reported by region.

The pattern of patient's individual FVIII consumption was also reported for Brazil (Table 3). Brazilian patients showed low rates of FVIII infusion when compared to the whole population. Variations for this outcome between the different regions studied were not as significant as for ABR. Brazilian patients relied mostly on standard half-life recombinant FVIII, while most patients in Africa received plasma-derived products.

**Table 3:** FVIII replacement therapy profile in Brazil.^4^

Variable	FVIII Replacement Product (IU/kg/year)	Pre-baseline mean (SD)	On-study mean (SD)	Total duration mean (SD)
Pre-baseline and on-study annualized FVIII utilization rates of the 6-month analysis	Overall (*n* = 41)	3325 (1526)	3457 (1612)	3396 (1546)
	Standard half-life only (*n* = 35)	3265 (1225)	3391 (1434)	3335 (1307)
	Extended half-life only (*n* = 3)	5925 (2299)	5795 (2234)	5851 (2262)
	Plasma-derived only (*n* = 0)	NA	NA	NA
	Combination of products (*n* = 3)	1421 (370)	1888 (269)	1663 (78.7)
Pre-baseline and on-study annualized FVIII infusion rates of the 6-month analysis	Overall (*n* = 41)	163 (60.0)	172 (63.1)	168 (60.2)
	Standard half-life FVIII only (*n* = 35)	170 (60.5)	177 (61.8)	174 (60.3)
	Extended half-life FVIII only (*n* = 3)	102 (19.1)	100 (19.4)	101 (19.3)
	Plasma-derived FVIII only (*n* = 0)	NA	NA	NA
	Combination of FVIII products (*n* = 3)	140 (42.1)	185 (77.1)	163 (54.3)

NA: Not applicable; FVIII: factor VIII.

Concerning the frequency of FVIII infusions, Brazil had the highest mean rate: pre-baseline: *n* = 163 (per year: 60.0); on-study: *n* = 172 (per year: 63.1); total study duration: *n* = 168 (per year: 60.2) of the regions which, considering FVIII utilization rates were low, implies that probably lower doses were used for each infusion when compared to other countries.

Data on adverse events were not reported separately by region, and overall adverse events were seen in 43.5 % of patients, although only 4.8 % were considered serious events (according to the Common Terminology Criteria for Adverse Events - CTCAE). No adverse event led to discontinuation of treatment.

**Figure 2:**
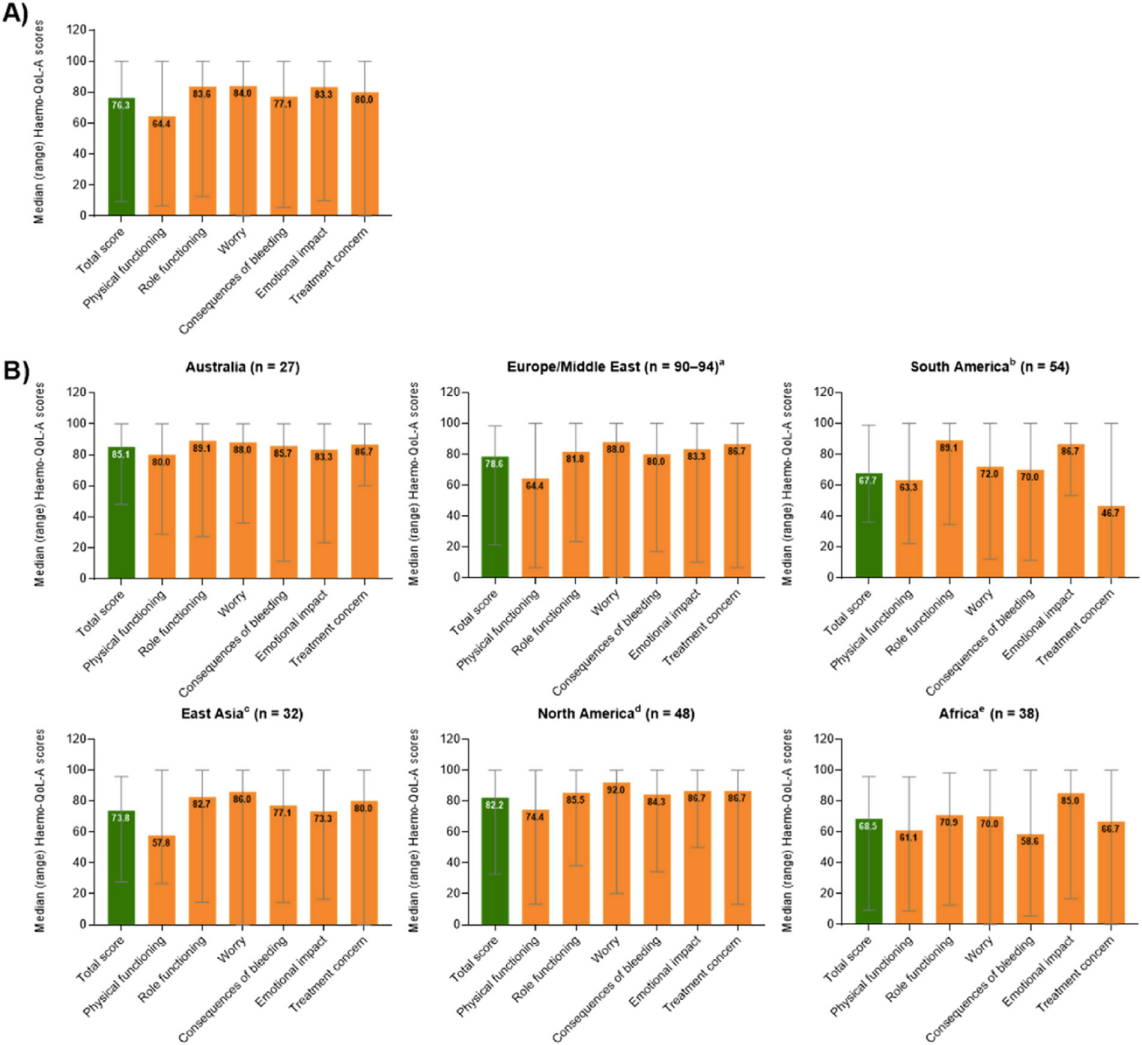
Median (range) overall transformed Hemo-QoL-A total and domain scores at baseline (A) for all participants globally (*n* = 298) and (B) for participants by region.^4^

Patient reported QoL outcomes (total and stratified by region) concerning the Hemo-QoL-A tool are depicted in Figure 2 (higher scores representing better health-related QoL). For Brazil, the highest domain scores were observed for emotional impact (86.7 points) and role functioning (89.1 points), while the lowest scores were observed for physical functioning (63.3 points) and treatment concern (46.7 points). Noticeably, the treatment concern domain (that assesses confidence of patients in respect to safety and accessibility to treatment, e.g. “I worry about the availability of hemophilia products”) for Brazilian patients was the lowest score among all the regions evaluated. Also, total score for Brazil fared unfavorably when compared to other countries with the lowest score observed (67.7 points). Results for the additional QoL scales applied were not reported separately for Brazil or other regions.

Upon discussion of the results, the authors argue that it is somewhat contradictory that countries and regions with such a low rate of FVIII utilization, such as Brazil and Africa, eventually presented with ABRs comparable to other regions, and especially such a low prevalence of problem joints (the lowest rates among the countries studied). Possibility underreporting should be considered. Another relevant drawback is the fact that this study enrolled patients that were motivated to take part in a gene therapy study that would follow this first 6-month observational follow up. As so, patients would probably be more prone to have a good adherence to treatment and to be dissatisfied with current therapeutic options in use. Site selection also could have influenced results as only facilities capable of providing structures demanded by gene therapy studies were selected.

### *Study by* Borges et al.*^7^*

This research, published only as an abstract, evaluated the impact of a pharmacokinetic-guided prophylaxis strategy for hemophilia A patients using the myPKFiT™ tool developed for alfa-octocog™ recombinant FVIII (Advate, Takeda). Effects in replacement costs and bleeding episodes were assessed. Men with hemophilia A due to a severe or moderate deficiency but without detectable inhibitors on current use of alfa-octocog were evaluated for enrollment at two Brazilian hemophilia treatment centers (in the states of Paraná and Minas Gerais).

The inclusion criteria were that patients should present ≥50 exposure days, age ranging from 1 to 65 years, weigh from 12 to 120 kg, have a bleeding-free period of at least 2 wk, with the last registered surgical procedure being ≥6 months before enrollment. The detection of inhibitors (>0.6 BU/mL at two time points) during follow up resulted in patient exclusion from the study.

All information pertaining anthropometric and hemophilia-related data were obtained using a standardized form and pharmacokinetics analysis by the myPKFiT™ software using a one-step test. This analysis guided dose adjustments based on bleeding phenotype, arthropathy, and physical exercise. The replacement regimen and FVIII utilization was evaluated before and after guided adjustments. Under 15-year-old patients were followed up for six months, while older patients were monitored for 12 months. ABR was calculated based on reported bleeding episodes.

A total of 37 patients were included. For the younger subgroup (*n* = 20), 75 % had severe hemophilia A and 65 % had no hemophiliac arthropathy (half of these were on primary prophylaxis). For those in the older subgroup (*n* = 17), 7 % were severe cases, one patient was treated exclusively on-demand before adjustment, none were on primary prophylaxis, and 12 % had no hemophiliac arthropathy. Three patients were excluded from the analyses: one due to development of inhibitors during the follow up, one transferred to on-demand only treatment, and one received prescriptions of plasma-derived FVIII after adjustments.

The median ABR for younger patients in this cohort was 3.0 (interquartile range: 0.5–10.0) before dose adjustment and 1.0 (interquartile range: 0.0–2.0) during the follow up. In the younger population, FVIII replacement costs increased after pharmacokinetics-guided adjustments (*p*-value <0.0001) mainly due to increased costs of prophylaxis (*p*-value <0.0001), while episodic therapy costs were reduced (*p*-value <0.05). For older patients, the ABR did not change significantly comparing before and after the intervention (values for rates were not reported). Although total treatment costs did not differ comparing before and after treatment adjustments, episodic therapy costs were reduced (*p*-value = 0.039).

### *Study by* Cerqueira et al. *– ahead study**^8^*

This study reports data from the International Anti-Hemophilic factor (recombinant) Hemophilia A outcome Database (AHEAD), a prospective, non-interventional, multicenter study (NCT02078427) designed to assess long-term effectiveness and safety of Anti-Hemophilic factor (recombinant) (rAHF) in patients with hemophilia A in the real-world clinical practice. Patients with moderate or severe hemophilia A (FVIII ≤5 %) were enrolled. Primary endpoint was joint health outcomes evaluated using the Gilbert score (pain: 0–3; bleeding: 0–3; physical exam: 0–12) or Hemophilia Joint Health Score (HJHS) according to hemophilia treatment center preferences. Secondary endpoints included ABR, annualized joint bleeding rates, and safety endpoints. This publication was presented as an abstract in the International Society on Thrombosis and Haemostasis (ISTH) Meeting and reports demographic and clinical characteristics at screening from the safety analysis set for patients in the AHEAD Brazil subset at the 6th interim analysis (cutoff date July 2019).

The Brazilian subset included 203 male patients with a median age of 13.0 years (range: 0–43 years). One hundred and ninety received prophylaxis (median age: 14.0; range: 0–43 years), two received on-demand treatment (median age: 12.0; range: 0–24 years), and 11 patients with inhibitors received immune tolerance induction (ITI; median age: 12.0; range: 3–34 years). In the 12 months prior to screening, bleeding events had occurred in 130 (68.4 %) patients on prophylaxis, one (50.0 %) on-demand patient, and four (36.4 %) patients receiving ITI. Computed median ABR for the 190 prophylaxis patients was 2.0 (range: 0.0–30.0), for the on-demand patients it was 5.0 (range: 0.0–10.0), and for the ITI patients it was 0.0 (range: 0.0–26.0). Results for other variables in the study can be found in Table 4.

**Table 4:** Outcomes in the Brazilian Anti-hemophilic factor Hemophilia A outcome database (AHEAD) subset of patients.^8^

Outcome	Prophylaxis	On demand	ITI
Mean Gilbert score (n)	35	–	1
Median (range)	1.0 (0.0–5.0)	–	1.0 (1.0–1.0)
HJHS: Global Gait Score (n)	86	0	8
Median (range)	1.0 (0.0–4.0)	–	1.0 (0.0–4.0)
AJBR (n)	190	2	11
Median (range)	1.0 (0.0–30.0)	4.5 (0.0–9.0)	0.0 (0.0–19.0)

ITI: immune tolerance induction.; HJHS: Hemophilia Joint Health Score; AJBR: annualized joint bleeding rate.

### *Study by* Ozelo et al. *– BRAVE**^9^*

This observational retrospective study aimed at collecting real-world evidence of Brazilian hemophilia A patients and was presented as an abstract on the 13th Annual Congress of European Association for Hemophilia and Allied Disorders. Three Brazilian Hemophilia treatment centers participated in data collection that was performed from January 2014 to December 2017. Outcomes of a total of 30 inhibitor patients (*I*+) and 60 non-inhibitor patients (I-) were reported.

Median age at enrolment was 18 (*I*+) and 26 (I-) years. Prophylaxis was used for 83.3 % of the *I*+ patients (with immune tolerance of 93.3 %) and 95 % of the I- patients. At least one bleeding episode was observed in 97.8 % of all patients. For the I- Group, the ABR was 2.98 (range: 2.15–3.8) with 10.17 % having an ABR of ≤3, while for the *I*+ Group, the ABR was 4.84 (range: 3.93–5.74) with only 3.33 % of patients having an ABR of ≤3. Additionally, FVIII prophylaxis and on-demand ABR were respectively 4.04 (range: 3.51–4.56) and 1.92 (range: 0.35–3.48), for the I- Group, and 6.72 (range: 5.7–7.74) and 3.93 (range: 1.44–4.46) for the *I*+ Group. Statistically significant differences in estimates were not reported. Authors state that results demonstrate significant healthcare resource utilization indicating that an improvement in Brazilian hemophilia A management strategies is needed.

### *Study by* Rodrigues et al.*^10^*

This abstract, presented in the 2016 World Congress of the World Federation of Hemophilia, reports a retrospective study evaluating the efficacy and FVIII concentrate consumption for daily tertiary prophylaxis in a group of severe hemophilia A adolescents (FVIII <1 % IU/dL) managed at the State University of Campinas referral center.

Enrolled patients should have been guaranteed a daily prophylaxis regimen as a modification from a previous replacement protocol. The ABR and monthly FVIII consumption rate from the period under daily prophylaxis was compared to the 12-month period previous to enrollment.

Six of 33 (18 %) adolescent patients received daily prophylaxis and were eligible for analysis. The median age was 14 years (range: 12–18). Previous regimen of enrolled patients was 15–23 IU/kg FVIII every other day (four patients) or 20 IU/kg twice or three times per week (two patients). During daily prophylaxis, patients received 500–1000 IU/day FVIII. Mean dose was 12.14 IU/kg (range: 7.8–16.9). At publication, patients had a median period under treatment of 16.33 months (range: 4–28) and all were still being treated in a daily prophylaxis regimen.

Observed ABR was 10.0 (range: 4.0–26.0) in the non-daily period and 1.7 (range: 0–8.5) with the daily prophylaxis regimen (*p*-value = 0.015). For annualized joint bleeds, rates of 4.98 (range: 2.04–24) and 0.42 (range: 0–6) were registered for non-daily and daily prophylaxis, respectively (*p*-value = 0.04). No significant difference was observed in monthly FVIII concentrate consumption between regimens (non-daily: 11,698 IU/month; range: 6500–20,416 IU/month; daily: 11,673 IU/month; range: 2833–23,979 IU/month; *p*-value = 0.94).

Summary of findings concerning ABR for Brazilian patients are shown in Table 5.

**Table 5:** Summary of ABR reported in eligible publications.

Study (Year)	n	Age (years) n	Baseline* ABR median (range)	Post-Intervention ABR median (range)	Setting
Kenet et al.^4^	41	27	0.8 (0–19.3)	NA	Adult-only patients. Considers six months of retrospective data added to at least six months of prospective follow up
Borges et al.^7^	37	≤15 = 20^†^ >15 = 17^†^	3.0 (0.5–10.0)	1.0 (0–2.0)	ABR reported only for the younger cohort. Improvement with myPKFiT™ tool statistical significance not reported
Cerqueira et al.^8^	190	14	2.0 (0–30.0)	NA	Results for prophylaxis cohort
Ozelo et al.^9^	60	26	4.04 (3.51–4.56)	NA	Results for non-inhibitor prophylaxis group
Rodrigues et al.^10^	6	14	10.0 (4.0–26.0)	1.7 (0–8.5)	Adolescent patients only. Conventional versus daily replacement (*p*-value = 0.015)

ABR: annualized bleeding rate; NA: not applicable.

⁎ Rates depicted here are those registered before intervention for patients on prophylaxis treatment. ^†^Number in each category.

### Quality assessment

A moderate risk of confounding was observed in three studies^8^, ^9^, ^10^ due to a lack of clear information about inclusion and exclusion criteria of the study participants; thus, it was not clear if confounding was successfully controlled at baseline. In addition, it was not clear if analyses were performed with appropriate statistical methods. All studies recruited consecutive patients that met screening criteria and were judged as low risk of bias in the selection of participants. As prophylaxis was the only evaluated intervention, misclassification of interventions was unlikely and did not apply to these studies. All studies were judged as low risk in respect to deviations from intended intervention domain as no co-interventions were addressed by the participants and no deviations from intended intervention were reported. The results of the studies were not biased by missing data as there was no incomplete data collection and no participant was excluded from the analyses. Finally, there was no selective reporting related to ABR outcome. A summary of quality assessment is shown in Table 6.

**Table 6:** Risk of bias summary for non-randomized clinical trials for prophylaxis in severe hemophilia A patients according to the ROBINS-I tool.

Author	Bias due to confounding	Bias in selection of patients into the study	Bias in classification of intervention	Bias due to deviations from intended interventions	Bias due to missing data	Bias in measurement of outcomes	Bias in selection of the reported result	Overall risk
Kenet et al.^4^	Low	Low	NA	Low	Low	Low	Low	Low
Borges et al.^7^	Low	Low	NA	Low	Low	Low	Low	Low

Cerqueira et al.^8^	Moderate	Low	NA	Low	Low	Low	Low	Moderate
Ozelo et al.^9^	Moderate	Low	NA	Low	Low	Low	Low	Moderate

Rodrigues et al.^10^	Moderate	Low	NA	Low	Low	Low	Low	Moderate

*A study was assigned low risk if the study was judged to be at low risk for all domains.

## Discussion

Treatment of severe hemophilia A has witnessed important steps towards a less immunogenic and more efficacious therapy over the last years. But, as a rare disorder, information on hemophilia A is usually scarce, especially real-world evidence. Brazilian data are no exception, and as a result, a very limited number of studies was retrieved for this systematic review regarding ABR, and no study correlating ABR with adherence to therapy was found. Also, it is noteworthy that data come mainly from the southern region of Brazil, limiting the scope of patients and probably favoring patients with improved access to healthcare facilities.

Apart from the scarce number of reports, quality of evidence was also considered moderately prone to bias in the majority of studies found. Although ROBINS-I is the tool indicated for risk assessment of non-randomized clinical trials, the use of this tool with the objective of evaluating ‘before and after’ interventions has not been validated yet. Thus, it is recommended that the qualitative assessment of each domain should be prioritized over the general results.

ABR for Brazilian non-inhibitor patients under conventional prophylactic treatment showed great variance with median values ranging from 0.8 to 10, in different population settings (Table 5). These estimates are grossly comparable to those observed in other regions as reported by Kenet et al.^4^ However, results from Kenet et al.^4^ may have been influenced by selection bias, with a possible underestimation of bleeding episodes due to a better treatment-compliant population.

However, it is known that, although ABR has been used by many contemporary studies as a default principal efficacy outcome, it suffers from great variability between hemophilia treatment centers.^11^ Estimation of bleeding rates poses a complex challenge and depends on a myriad of patient-related and extrinsic factors, such as the individual clotting factor level, pharmacokinetic profile and pain perception, the subject's age, health status, activity level, dosing regimen, bleeding event definition, follow-up time, and number of patients analyzed. ABR estimation is prone to subjective assessment, as patients and physicians are required to define each bleed.^11^

Indeed, additional data reported by the studies retrieved deserve a special mention. First, Kenet et al.^4^ showed that access to treatment is a major concern for Brazilian hemophilia A patients, which may reflect previous difficulties in receiving timely and adequate infusions of FVIII. Also noteworthy, patients in Brazil, differently from other countries studied by Kenet et al.^4^, mainly have access to standard half-life products (>85 % of patients in the cohort) and demonstrate a lower comparative FVIII utilization rate; this could be evidence of inadequate adherence. Furthermore, studies by Borges et al.^7^ and Rodrigues et al.^10^ demonstrated that maintaining more stable and continuous levels of FVIII activity effectively reduce the ABR, at least for one subgroup of patients. Such a premise has been for a long time the main core of many initiatives in the development of therapeutic options for hemophilia A, aside from the efforts on reducing immunogenicity of replacement factors.^12^ However, efficacy of such replacement regimens demanding frequent factor infusions pose a significant burden upon patients, compromising long-term effectiveness, treatment adhesion and QoL. Also, financial costs increase as more infusions are required to maintain a lower ABR. As a recent alternative addressing such obstacles, gene therapy has emerged as a promising pathway of treatment in the near future.^13^^,^^14^

## Conclusion

Available information on efficacy of severe hemophilia A management in Brazil currently relies on scarce and possibly biased information. It should be strongly emphasized that Brazil is in great need of a structured and coordinated effort towards better collection, analysis and reporting of data of severe hemophilia A patients. Overcoming the scarcity of information about this specific topic is key to maintain improvement in policies directed toward Brazilian hemophilia A patients.

Despite of this, one could infer that the great variance in ABR in different studies, potential selection bias of patients (with better access to healthcare facilities and more compliant to treatment) and the lower comparative FVIII utilization rate suggest that Brazilian non-inhibitor patients still need better treatment.

## Conflicts of interest

None.
